# Editorial: To Know or Not to Know: causes and evolution of lack of awareness of cognitive decline in neurodegenerative diseases, volume II

**DOI:** 10.3389/fnagi.2025.1568173

**Published:** 2025-02-12

**Authors:** Federica Cacciamani, Kristy A. Nielson, Bernard J. Hanseeuw, Geoffroy P. Gagliardi, Patrizia Vannini

**Affiliations:** ^1^ARAMISLab, Institut du Cerveau - Paris Brain Institute - ICM, Sorbonne Université, CNRS, Inria, Inserm, AP-HP, Hôpital de la Pitié Salpêtrière, Paris, France; ^2^Qairnel SAS, Paris, France; ^3^Department of Psychology, Marquette University, Milwaukee, WI, United States; ^4^Department of Neurology, Medical College of Wisconsin, Milwaukee, WI, United States; ^5^Department of Radiology, Massachusetts General Hospital, The Gordon Center for Medical Imaging, Boston, MA, United States; ^6^Department of Neurology, Cliniques Universitaires Saint-Luc, Université Catholique de Louvain, Brussels, Belgium; ^7^Brigham and Women's Hospital and Harvard Medical School, Boston, MA, United States; ^8^Massachusetts General Hospital and Harvard Medical School, Boston, MA, United States

**Keywords:** anosognosia, subjective cognitive decline, Alzheimer's disease, self-awareness, denial, neuroimaging, biomarkers

## Introduction

Anosognosia, or the lack of awareness of cognitive, behavioral, or functional deficits, represents a critical challenge in the clinical management of neurodegenerative diseases such as Alzheimer's disease (AD) and frontotemporal dementia. This condition not only impacts patients, caregivers, and healthcare systems (D'Souza et al., 2011; Turró-Garriga et al., 2013) but also raises important questions about its underlying mechanisms and evolution (Hanseeuw et al., 2020; Starkstein, 2014; Vannini et al., 2017). Despite its critical importance, significant gaps remain in our understanding of cognitive self-awareness in neurodegenerative diseases. Building on the momentum of the first edition of To Know or Not to Know, this second volume continues to explore these pressing issues through innovative approaches and multidisciplinary perspectives. The eight articles in this volume span a diverse range of themes and methodologies, reflecting the multifaceted nature and importance of awareness research.

## The link between subjective cognitive decline, hypernosognosia, and anosognosia

Recent research, including findings from Volume I of this Research Topic (Chapman et al., 2022), suggests that subjective cognitive decline (SCD), hypernosognosia, and anosognosia may represent distinct yet interconnected states of awareness in AD. SCD refers to self-reported cognitive complaints, which may or may not be accompanied by objective cognitive decline (Jessen et al., 2020). The concept has been the subject of debate within the field, wherein SCD has been demonstrated to be prone to biases and may be non-specific for neurodegenerative processes (Cacciamani et al., 2023). Given the heterogeneity of self-reported cognitive complaints and their possible association with factors such as mood, comorbidities, and lifestyle, SCD may benefit from refined characterization and stratification to enhance its predictive value for neurodegenerative processes. This would allow for a clearer distinction between individuals whose complaints are driven by early AD pathology vs. those influenced by non-pathological factors. For example, there may be specific subgroups where SCD is more strongly linked to underlying AD pathology. Ben-Ami et al. provide compelling evidence for this hypothesis. In cognitively normal middle-aged individuals with a family history of AD and the Apolipoprotein-E (APOE) ε4 genotype, higher subjective cognitive decline (SCD) was associated with disrupted white matter integrity, notably in the uncinate fasciculus, and abnormal brain activity, specifically elevated frontal activity, during a working memory task. These findings corroborate a recent study showing that ε4 carriers had significantly poorer self-awareness of their objective executive functioning than comparable non-carriers (Evans et al., 2024). Taken together, these findings suggest that functional neural alterations may index early AD pathology in those exhibiting altered neural mechanisms related to self-perceived cognitive decline, particularly in ε4 carriers, even in the absence of detectable objective deficits. While SCD and anosognosia differ in clinical presentation, alterations in self-monitoring processes may point to similar mechanisms being triggered as AD pathology progresses. Indeed, even during resting-state measures, anosognosia has been linked to disrupted neural network functioning. Using resting-state fMRI, Tondelli et al. found anosognosia to be associated with decreased intrinsic functional connectivity in the default mode network and increased connectivity in the salience network. They suggest that this “imbalance” between self-referential networks may contribute to anosognosia, even in the absence of direct structural damage.

Hypernosognosia, on the other hand, refers to heightened self-awareness of cognitive changes (Vannini et al., 2017), marked by cognitive complaints that are not corroborated by informants or objective assessments. Studies suggested that in the early stages of AD, hypernosognosia may give way to anosognosia as the disease progresses, with awareness declining further during the prodromal phase and dementia onset. López-Martos et al. provided further evidence in this Research Topic. Among cognitively normal individuals reporting complaints, hypernosognosia was associated with higher amyloid-beta levels. Conversely, in individuals without complaints, awareness followed a non-linear trajectory, increasing up to a specific amyloid-beta threshold before declining thereafter, consistent with previous findings (Gagliardi et al., 2020).

## Implications of anosognosia for caregivers

Caregiving in AD presents unique challenges, as caregivers often shoulder the dual burden of managing the patient's evolving cognitive and functional decline while coping with their own emotional and physical strain (Starkstein et al., 2007). Anosognosia exacerbates these challenges by limiting the patient's ability to recognize their deficits, leading to increased caregiver stress and complicating care strategies (Turró-Garriga et al., 2016). In a large cohort of cohabitating spouse-patient dyads where the patient was diagnosed with dementia, Alexander et al. highlighted these issues showing that caregiver reports of their spouse's cognitive difficulties were greater than the spouse's own reports. Lower concordance between patient and caregiver assessments was significantly associated with reduced patient autonomy and increased levels of perceived caregiver stress. Similarly, Marks et al. showed participants with poorer awareness of their performance during tasks involving planning were more dependent on activities of daily living (ADL) and exhibited worse cognitive function. These findings underscore the importance of assessing awareness to anticipate caregiving needs and plan for appropriate interventions. Relatedly, Wang et al. found that individuals with MCI and anosognosia from the ADNI cohort developed neuropsychiatric symptoms earlier than those without anosognosia. Relevant symptoms included delusions, hallucinations, agitation, apathy, disinhibition, irritability, and aberrant motor behavior. Anosognosia may thus increase vulnerability to neuropsychiatric symptoms and highlight the need for caregiver support and services.

## Distinguishing anosognosia from denial

Anosognosia and denial, though distinct, can appear deceptively similar in clinical practice, often complicating diagnosis and care. Differentiating the two is crucial for developing appropriate therapeutic strategies, understanding patient behaviors, and ensuring that interventions are both effective and compassionate. Prigatano et al. explored the distinction between anosognosia and denial through case studies, defining anosognosia as a neurological inability to recognize cognitive deficits, and denial as a psychological defense mechanism. The authors proposed a scoring system to differentiate these conditions, which involves characterizing denial as (1) involving emotional responses such as anxiety, agitation, or anger when difficulties are discussed; (2) the ability to recognize when behaviors affect others, which is not consistent with anosognosia; and (3) actively resisting the acknowledgment of deficits through counterarguments or avoiding discussion.

## The use of virtual reality to improve self-awareness in AD

Interventions to address anosognosia in AD remain limited, despite the profound impact this condition has on disease management and patient outcomes. Traditional therapeutic approaches have shown little success in improving self-awareness, underscoring the need for innovative methods. In this context, Latgé-Tovar et al. proposed virtual reality as a promising tool for both the assessment and improvement of self-awareness, including disease awareness, in AD. They propose the creation of immersive and ecological experiences based on perspective-taking. The authors reviewed the possibilities and potential limitations of this approach and highlighted the need for further research to validate its efficacy and applicability.

## Conclusion and future directions

While this important second Research Topic advances our understanding of anosognosia in AD, we acknowledge that significant gaps remain. Notably, the scarcity of longitudinal data limits our ability to fully map how awareness changes in individuals over time, and how it relates to the emergence to AD pathology and other clinical symptoms. Similarly, more research is needed to refine diagnostic tools, develop effective interventions, and explore the interplay between biological, psychological, and social determinants of awareness.

We hope this Research Topic inspires further exploration into this critical area and serves as a resource for researchers and clinicians dedicated to advancing the field of aging neuroscience.
